# Epidemiological profiles and causes of sudden deaths of various ages in Ethiopia: an autopsy-based study

**DOI:** 10.12688/f1000research.142511.1

**Published:** 2023-11-07

**Authors:** Alemayehu Shiferaw Lema, Sesen Tsegaye Tekle

**Affiliations:** 1 Department of Forensic Medicine and Toxicology, St. Paul's Hospital Millennium Medical College, Addis Ababa, Ethiopia, 1271, Ethiopia

**Keywords:** Sudden death, Sudden and unexpected natural death, Sudden cardiac death, Causes of death, Autopsy, Natural death, Ethiopia

## Abstract

**Background:**

Sudden death is an important global public health issue. An autopsy is an important source of epidemiological data, as the considerable causes of sudden death remain hermetic until postmortem examination. This study is devoted to evaluating the sociodemographic, behavioral, clinical and pathological characteristics of sudden deaths of various ages in Ethiopia.

**Methods:**

This is an observational, prospective, descriptive study that included all sudden deaths observed over 1 year at St. Paul’s Hospital and Millennium Medical College, Addis Ababa, Ethiopia.

**Results:**

Sudden death (n = 568) accounted for 11.5% of all autopsied cases. There were 482 males and 86 females (M:F ratio of 5.6:1) and a mean age of 44.81±17.349. The peak age group was the fourth and fifth decades, accounting for 43.9% of the cases. Chronic substance abuse and a history of prior illness were declared in 40.1% and 38% of victims of sudden death, respectively. Cardiovascular (36.1%), respiratory (32.6%), and gastrointestinal system (19.5%) pathologies were the most common causes. The leading underlying causes of sudden death were ischemic heart disease and pneumonia. Most (86.6%) sudden deaths occurred outside of a hospital setting.

**Conclusions:**

Most of the causes of sudden death in Ethiopia can be prevented and treated. The majority of sudden deaths are silent without preexisting symptoms. Therefore, it is vital to develop public health measures that will help educate the community about the importance of recognizing the manifestation of various clinical conditions and the need to seek immediate clinical help. Furthermore, efforts should be made to make healthcare facilities accessible and affordable with adequate diagnostic and management capacity. Documentation of autopsy-based data could provide important epidemiological information to guide medical services, prevention efforts, and control measures.

## Introduction

Sudden death is an important global public health issue, especially when it occurs in apparently healthy individuals due to the bearing these losses have on individuals, families, communities and wider society. The definition of sudden death varies according to authorities and conventions. ^1^ According to the World Health Organization (WHO), death is considered a sudden death (SD) when a nonviolent and unexpected death occurs within 24 hours after the commencement of a fatal illness. ^2^ The frequency and pattern of sudden death in different parts of the world vary greatly due to the diversity of prevalent diseases in various nations and due to diverse genetic and environmental factors. ^3^^,^^4^

Sudden unexpected natural death is often the initial clinical appearance of an underlying condition in people who had previously been asymptomatic and seemingly healthy. ^3^^,^^5^ Furthermore, since many of these deaths are unwitnessed and unattended by clinicians, the extent of the problem is difficult to determine. In this circumstance, an autopsy is an initial and sole opportunity to determine and document the precise cause of death. ^1^^,^^4^^,^^6^ In Ethiopia, SD is regarded as a type of medicolegal death for which an autopsy is required to determine the cause.

Public health authorities, therefore, depend heavily on vital statistics systems with a focus on the cause of death (COD) for formulating their programs, both curative and preventive specialist services. Mortality statistics are an essential component of the nation's vital statistics system. They are the ingredients to measure population growth and provide a demographic perspective for the planning of development in social services. Cause-specific mortality rates are crucial measures of population health trends. There are several challenges in interpreting epidemiological information in resource-limited settings, including a lack of uniformity and low quality of death certificates as well as the utilization of verbal autopsies. ^6^^–^^8^ Thus, vital health information may not reach the national registry, which could prevent the implementation of crucial interventions and prevention measures. Accurate evidence on causes of death is necessary to recognize the general epidemiological profile of diseases in Ethiopia and to support decision-makers in prioritizing the public health agenda. An autopsy is an important source of epidemiological data, as the considerable causes of SD remain hermetic until postmortem examination. ^1^ Therefore, epidemiological autopsy-based data on sudden death causes are vital to understand the characteristics of the affected population and customize public health programs. To our knowledge, this is the first study paper on SD in Ethiopia and is devoted to documenting the sociodemographic, behavioral, clinical and pathological characteristics of sudden death victims of various ages.

## Methods

This is an observational, prospective, descriptive study that included all sudden unexpected natural cases observed from July 1, 2019, to June 30, 2020 **,** in the Department of Forensic Medicine and Toxicology of St. Paul’s Hospital and Millennium Medical College (SPHMMC), Addis Ababa, Ethiopia. SPHMMC is a tertiary hospital and medical college governed by a board under the Federal Ministry of Health. SPHMMC in Addis Ababa and Aider Hospital in the Tigray region were the only two centers in the country providing forensic medicine and toxicology services during the study period. The Department of Forensic Medicine and Toxicology, SPHMMC, offers postmortem services to almost all regions of Ethiopia, except cases from the Tigray region served by Aider Hospital.

This study included all SD cases (according to the WHO definition) brought for autopsy from July 1, 2019, to June 30, 2020. The WHO defines SD as death that occurs within 24 hours after the disease’s commencement in a person not known to have been diagnosed with a serious disease, accident, or poisoning. ^2^ In consideration of this definition, two factors were considered: 1) the time of onset of terminal signs or symptoms until death and (2) the expectation of death at the time of occurrence. The definition of the onset of terminal signs or symptoms used in this study was the time when an individual had to change his activity because of the illness. The time of death was defined as the time an individual was pronounced dead. For unwitnessed deaths in which an individual was known to have been alive within 24 hours before the pronounced time of death, they were considered sudden death. The second factor in deciding whether or not the death was sudden was the unexpected nature of the occurrence. The degree of disability reported before death was used as a measure of the expectation of death at the time of the event. Individuals who died but were not confined to their homes, health facilities, or other related institutions due to illness for more than 24 hours before death were not considered unexpected deaths and were not included.

Principal investigators and five well-trained medical doctors collected the data. Information on the biodata of the victims, clinical data and circumstances of the death was collected from all potential sources, such as police files, medical records, and direct interviews with the eyewitnesses, relatives, and friends of the victims. The duration of hospital stay before death was extracted from the hospital record, autopsy referral papers, and police requests.

Full postmortem examinations were carried out in each instance using the Letulle evisceration procedure, systematically inspecting all cavities, including the cranial, cervical, thoracic, and abdominal cavities. Organs were dissected, examined and checked for signs of gross pathological changes and violence. Autopsied specimens (whole organs or organ pieces) were fixed in 10% formalin for histopathological examination. Multiple sections with thicknesses of 4-5 mm were taken. The tissues were processed, subjected to paraffin sectioning at a thickness of 4 micrometers and then stained using hematoxylin and eosin staining.

A structured data collection form was used to obtain all relevant clinical, epidemiological and pathological data. The predesigned data collection form was pretested in 50 cases to maintain data quality, and the necessary amendment was made to the form before the actual data collection. Data entry was performed using Microsoft Excel 2016 and exported to the Statistical Package for Social Science (SPSS window version 26) for analysis. Descriptive summary measures were used to characterize sociodemographic, behavioral, clinical characteristics and circumstances of death. A chi-square test was used to compare categorical variables, and the level of significance was set at a p-value <0.05.

Ethics approval was obtained from the SPHMMC Institutional Review Board (Ethical clearance reference no: PM 23/188). The study was conducted as per the Declaration of Helsinki. All information was treated anonymously and confidentially using unique identification codes rather than individual names and identifications.

## Results

### Epidemiological characteristics

A total of 4,942 medicolegal autopsies were performed during the study period, of which 568 cases were due to SD, accounting for 11.5% of the total autopsied cases. The youngest case was a 1-day-old male newborn, and the oldest was a 98-year-old man, with a mean age of 44.81±17.349. Males (n = 482/586) were predominant over females (n = 86/586) at a ratio of 5.6:1. ^27^

**Figure 1.**
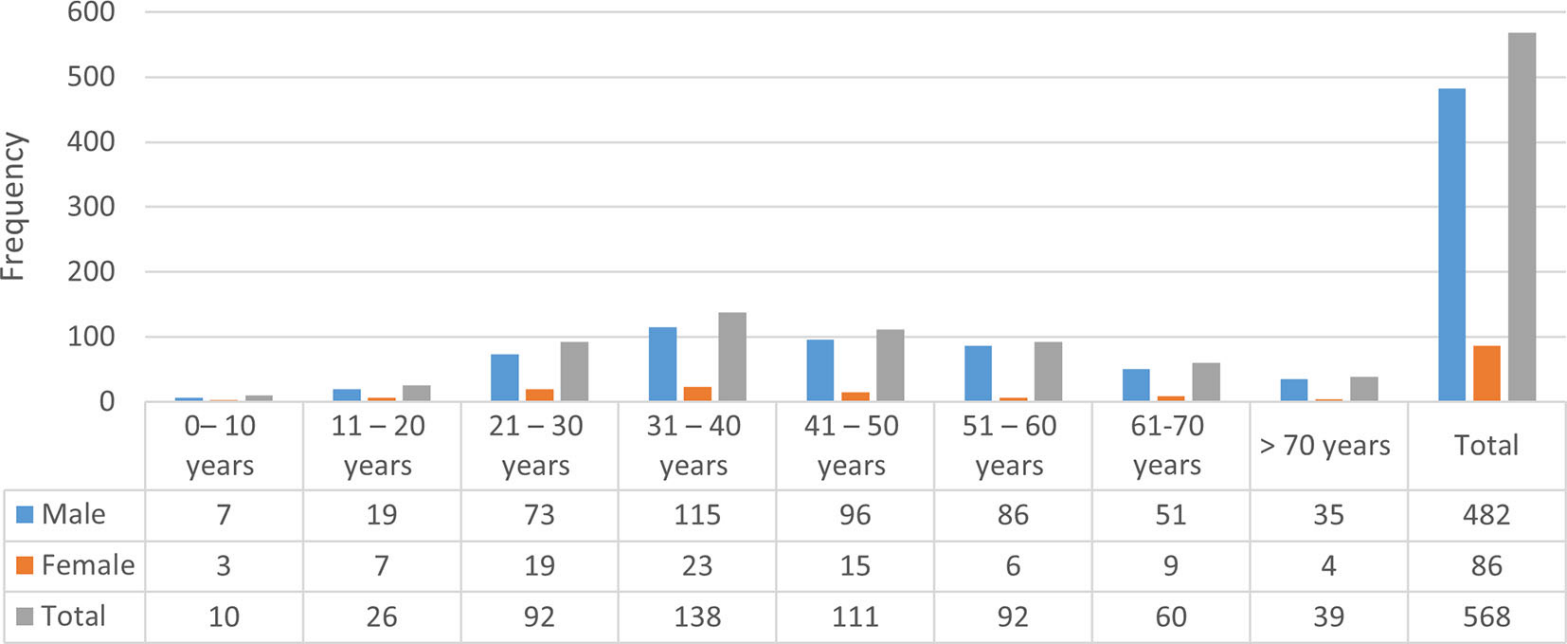
Age and sex distribution of sudden death cases (n=568).

The maximum number of sudden deaths (24.3%) was in the age group of 31-40 years, followed by 41-50 years, which represents 19.6% of all SD cases. SD was less prevalent in extreme age groups, less than 10 years (1.8%) and over 70 years (6.7%). Males outnumbered females in all age groups. The fourth decade was the most predominant age group seen in males and females, accounting for 23.9% and 26.7% of total sex-specific cases, respectively. The number of cases by age and sex is summarized in Figure 1.

More than two-thirds of the victims of SD (70%) belonged to urban areas, whereas 30% of victims were from rural areas. There was a statistically significant difference between the residence of the victims and sex (p<0.001). Most of the victims were from Addis Ababa city, the capital city of Ethiopia, accounting for 59.2% (n=336) of cases, followed by the Oromia region (23.6%), and the remaining 17.2% were from six other regions of the country. Table 1 summarizes the sociodemographic characteristics of the victims of sudden death.

**Table 1.** Summary of sociodemographic characteristics of sudden death victims.

Sociodemographic characteristics	Category	Frequency	Percentage
**Sex**	Male	482	84.9
	Female	86	15.1
**Residence**	Urban	397	70.0
	Rural	171	30.0
**Region/Administrative city**	Addis Ababa	336	59.2
	Oromia	134	23.6
	SNNPRS *	54	9.5
	Amhara	28	4.9
	Diredawa	7	1.2
	Harar	4	0.7
	Afar	3	0.5
	Gambela	2	0.4
**Occupation**	Not applicable (<18 years/student)	37	6.5
	Employed	325	57.2
	Unemployed	49	8.6
	Retired	5	0.9
	Unknown	152	26.8
**Education**	Preschool	10	1.8
	Illiterate	68	12.0
	Primary school	22	3.9
	Middle school	69	12.1
	High school	165	29.0
	Graduate/more	68	12.0
	Unknown	166	29.2
**Marital Status**	Unmarried	103	18.1
	Married	237	41.7
	Divorced	15	2.6
	Widowed/r	57	10.1
	Unknown	156	27.5
**Religion**	Orthodox	181	31.9
	Protestant	141	24.8
	Muslim	85	15.0
	Catholic	11	1.9
	Unknown	150	26.4

* SNNRS-Southern Nations Nationalities and Peoples’ Regional States.

### Clinical characteristics and habits of substance abuse data

Substance use was reported in approximately 40.1% (n=228) of all SD cases. Chronic alcohol use was reported in 24.3% of victims, followed by khat (Catha edulis) and cigarettes in 9.3% and 6.5%, respectively. Moreover, 10% of the victims use two or more of these substances. There was a statistically significant relationship between substance abuse and sex (p<0.001) and residence (p=0.004). A different distribution was observed for chronic alcohol use and sex (p<0.001).

More than one-third of cases (38%) had a history of prior chronic medical illness, and diabetes mellitus (n=97 cases, 17.1%) was the most common disease. In 5.8% of victims, there is a history of SD in first-degree relatives. There was a statistically significant relationship between the presence of a prior chronic illness and sex (p=0.022). Table 2 summarizes the clinical characteristics and habits of substance use and SD in first-degree relatives of victims.

**Table 2.** Clinical characteristics and habits of substance abuse data in sudden death cases.

Clinical and habit data	Frequency	Percentage
History of prior illness lifetime	216	38
Diabetes Mellitus	97	17.1
Hypertension	78	13.7
Ischemic heart disease	4	0.7
Valvular heart disease	4	0.7
Unspecified cardiac disease	1	0.2
Asthma	7	1.2
Chronic obstructive pulmonary disease	4	0.7
Liver disease	12	2.1
Peptic ulcer disease	3	0.5
HIV	4	0.7
Chronic kidney disease	2	0.4
History of sudden death first-degree relative	33	5.8
Unspecified cardiac disease	13	2.3
Ischemic heart disease	3	0.5
Asthma	10	1.8
Chronic obstructive pulmonary disease	3	0.5
Cerebrovascular accident	4	0.7
Substance abuse	228	40.1
Alcohol	138	24.3
Cigarette	37	6.5
Khat	53	9.3
More than one abovementioned substances	57	10
Last medical visit	568	100
Days	107	18.8
Weeks	86	15.2
Months	118	20.8
Years	16	2.8
Unknown	241	42.4

### Circumstances of sudden death

More than three-quarters of the victims were found dead (68%, n=387/568 cases), followed by victims who died at the time of arrival or after a short period of arrival in health facilities (23.1%, n=131/568 cases). Approximately 6.3% (n=36 victims) died instantaneously. Prodromal signs and symptoms were reported in 178 victims of SD. Of these 178 victims, 70.8% (n=126) sought medical help. The most common terminal signs and symptoms of SD were chest pain (n=45 cases), followed by dyspnea (n=43 cases) and syncope (n=42 cases). Of the 178 victims, 42.1% died within 1 hour of the onset of symptoms, while 36% and 21.9% of the victims died between 1-6 hours and 6-24 hours of the onset of symptoms, respectively. The majority of sudden death incidents occurred at home in 34.9% (n=198) of the cases, followed by public places and health facilities in 30.3% and 23.4%, respectively.

### Causes of sudden unexpected deaths

Cardiovascular system diseases (CVS) were the leading causes of SD, accounting for 36.1% of all SD cases. This was followed by respiratory and gastrointestinal system pathologies, accounting for 32.6% and 19.5%, respectively. Central nervous system (CNS) and genitourinary system (GUS) pathologies were the least prevalent causes of death, accounting for 10.6% and 1.2%, respectively.

Male cases were affected more than female cases in each system, except in the GUS, in which female cases were prevalent. There was a statistically significant difference between the causes of death by organ system and sex, p<0.004. Comparisons of COD by organ system and sex are summarized in Table 3.

**Table 3.** Comparisons of COD by organ system and sex.

System	Number of deaths			Percentage of all deaths	p-value
	Male	Female	Total		
Cardiovascular system	176	29	205	36	0.017 *
Respiratory system	160	25	185	32.6	0.003 *
Gastrointestinal system	97	14	111	19.5	0.474
Central nervous system	47	13	60	10.6	0.414
Genitourinary system	2	5	7	1.2	0.008 *
Total	482	86	568	100	<0.001 *

* Significant.

Ischemic heart disease (IHD) and pneumonia are the most common underlying causes of SD. The underlying COD in each organ system is detailed in Table 4. Pneumonia was the most common cause of sudden death in the first, third, and fourth decades of life and the second leading COD after the fifth decade following IHD. Ischemic heart disease was the most common COD in general and in both sexes, was the leading cause after the fifth decade and was the second most common COD in the age group of 21-40 years. Table 5 summarizes the common causes of SD by age group.

**Table 4.** Underlying COD distributed by system and sex.

System	Number of deaths			Percentage of all deaths
	Male	Female	Total	
**Cardiovascular system**	**176**	**29**	**205**	**36.1**
Ischemic heart diseases	124	19	143	25.2
Cardiomyopathies	29	1	30	5.3
Rheumatic valvular heart diseases	11	7	18	3.2
Ruptured aorta aneurysm	6	1	7	1.2
Infectious diseases (pericarditis and myocarditis)	6	1	7	1.2
**Respiratory system**	**160**	**25**	**185**	**32.6**
Pneumonia	95	11	106	18.7
Tuberculosis	33	3	36	6.3
Asthma	8	7	15	2.6
Chronic obstructive pulmonary disease	11	1	12	2.1
Pulmonary embolism	5	1	6	1.1
Lung abscess	3	2	5	0.9
Respiratory tract carcinoma	5	0	5	0.9
**Gastrointestinal system**	**97**	**14**	**111**	**19.5**
Liver diseases	54	5	59	10.3
Liver abscess	2	0	2	0.4
Pancreatitis	34	8	42	7.4
Pancreatic abscess	2	1	3	0.5
Perforation of gastric/duodenal ulcer	3	0	3	0.5
Intestinal perforation	1	0	1	0.2
Intestinal tuberculosis	1	0	1	0.2
**Central nervous system**	**47**	**13**	**60**	**10.6**
Subarachnoid hemorrhage	23	6	29	5.1
Spontaneous intraparenchymal hemorrhage	14	5	19	3.3
Infectious diseases (meningitis and brain abscess)	8	1	9	1.6
Infarction	1	0	1	0.2
Hydrocephalus with meningomyelocele	1	0	1	0.2
Space Occupying lesion	0	1	1	0.2
**Genitourinary system**	**2**	**5**	**7**	**1.2**
Pregnancy-related causes	0	5	5	0.9
Kidney diseases (perinephric abscess)	2	0	2	0.4
**Total**	**482**	**86**	**568**	**100.0**

**Table 5.** The most common causes of sudden death by age group.

Age group in years (frequency of sudden death cases)	The most common cause of sudden death	Frequency of cases (percentage)
0-10 (n = 10)	Pneumonia	7(70)
	Meningitis	2(20)
11-20 (n = 26)	Pancreatitis	7(26.9)
	Rheumatic valvular heart diseases	4(15.4)
21-30 (n =92)	Pneumonia	16(17.4)
	Ischemic heart diseases	14(15.2)
	Pancreatitis	12(13)
	Cardiomyopathies	10(10.9)
31-40 (n =138)	Pneumonia	27(19.6)
	Ischemic heart diseases	26(18.8)
	Liver diseases	15(10.9)
41-50 (n =111)	Ischemic heart diseases	31(27.9)
	Pneumonia	20(18)
	Liver diseases	16(14.4)
51-60 (n = 92)	Ischemic heart diseases	33(35.9)
	Pneumonia	14(15.2)
	Liver diseases	12(13)
61-70 (n = 60)	Ischemic heart diseases	18(30)
	Pneumonia	11(18.3)
	Chronic liver diseases	10(16.7)
	Cardiomyopathies	7(11.7)
71-98 (n = 39)	Ischemic heart diseases	18(46.2)
	Pneumonia	8(20.5)

The most common COD among CVS was ischemic heart disease, accounting for 69.8% of CVS deaths and 25.2% of all sudden deaths, that is, the leading cause of SD overall and in both sexes. Cardiomyopathy was the second most common COD in the CVS category and caused 5.3% of all sudden deaths ( Table 4). Only 4.4% of victims among these categories of sudden deaths had previously been diagnosed with cardiovascular disease.

In the cardiovascular category of sudden death, the modifiable cardiovascular risk factors (CVRFs) identified were diabetes mellitus in 31.7% (65/205) and hypertension in 17.6% (36/205) of the victims. Furthermore, a history of alcohol, khat, and smoking was declared in 45 victims. Chronic alcohol use was the most common substance used and was reported in 38 (18.5%) of 205 victims. This was followed by the use of khat and cigarettes, each of which was reported in 14 cases (6.8%).

The most common terminal symptoms reported among CVS victims of sudden death were syncope (n=39 cases) and chest pain (n=25 cases). In 61 of 205 cases (29.8%), there were no previous symptoms, and SD was the first manifestation of sudden cardiac death. There was a statistically significant difference between the CVS causes and sex (p=0.019).

The most common respiratory pathology was pneumonia in 57.2% of respiratory cases and 18.7% of all sudden deaths, that is, the second most common underlying cause of SD in general and in both sexes. This was followed by tuberculosis in 19.5% of respiratory cases and 6.3% of all sudden deaths ( Table 4). A history of substance abuse was reported in 24.8% (n= 46 victims) of 185 victims of sudden death from respiratory pathology. Chronic alcohol use was the most common substance used and was reported in 37 (20%) of 185 victims. This was followed by smokers and chronic khat use, each reported in 13 cases. The most common terminal signs and symptoms reported among respiratory causes of sudden death were dyspnea (n=34 cases, 18.4%) and chest pain (n=20 cases, 10.8%).

Gastrointestinal system (GIS) pathologies were the third leading cause of SD, occurring in 97 (19.5%) cases. Liver and pancreatic diseases were the two most common causes of sudden death among GIS pathologies, accounting for 53.2% (n=59 cases) and 37.8% (n=42 cases), respectively ( Table 4). A history of substance use was reported in 46.8% (n= 52) of 111 victims of sudden death from GIS pathology. Chronic alcohol use was the most common substance used and was reported in 45% (n=50/111) of victims, followed by khat and cigarettes in 19.8% and 4.5% of victims, respectively. The most common terminal signs and symptoms reported among the GIS causes of sudden death were abdominal pain (n=24 cases, 21.6%) and vomiting (n=7 cases, 6.3%).

CNS pathologies were the fourth most common cause of SD, occurring in 10.6% (n=60 cases) of all sudden deaths. Subarachnoid hemorrhage and spontaneous intraparenchymal hemorrhage were the two common CNS pathologies, which occurred in 48.3% (29/60) and 31.7% (5/22) of cases, respectively ( Table 4).

The least common cause of sudden death was related to GUS, which accounted for 1.2% of all sudden deaths. There was a statistically significant difference between the pathologies of GUS and sex, where p=0.48. There was a female preponderance of 71.4% and a mean age of 32.14±18.614 years. Pregnancy-related sudden deaths were the main cause of death in this group, occurring in five cases. Three of them died from obstetric bleeding (two cases of postpartum hemorrhage and one case of antepartum hemorrhage). A postpartum hemorrhage occurred after delivery at home in a rural area. Two cases of SD were due to rupture of ectopic pregnancy in 15- and 22-year-old single women, in which the family did not know the pregnancy status of the victims.

## Discussion

This is the first study article on sudden deaths in Ethiopia and is devoted to evaluating the sociodemographic, behavioral, clinical and pathological characteristics of 568 sudden deaths of various ages in Ethiopia. The frequency and pattern of sudden death in different parts of the world vary greatly due to the diversity of prevalent diseases in various nations and due to various genetic and environmental factors. ^3^^,^^4^ The present study reveals that sudden unexpected death with a known natural cause of death constitutes approximately 11.5% of all autopsy cases. This finding matches those of other developing countries. ^3^ This finding is inconsistent with that reported in developed countries. ^5^^,^^9^ It is challenging to compare the magnitude of SD in different parts of the globe because it varies mainly as a function of the diversity of prevalent diseases in various nations and due to diverse genetic and environmental factors. ^3^^,^^4^ Furthermore, various definitions of SD, inclusion criteria and age groups that were evaluated in the study all contribute to the variation in SD incidence described in different studies.

The findings revealed a mean age of 44.8117.349 years. This is consistent with the studies conducted in Nigeria and Libya. ^3^^,^^4^^,^^10^ The maximum number of sudden death cases (24.3%) was in the age group of 31-40 years, followed by 41-50 years, representing 19.6% of all SD cases. This finding matches various studies. ^11^^–^^13^ All of these studies noted that middle age groups of 30 to 50 years are at high risk for SD. This finding appears to reflect behavioral and environmental factors that impact the health of society resulting from urbanization, the shift to a Western lifestyle, and the rapid nutritional transition and sedentary lifestyle with increased substance abuse habits.

Of 224 sudden death cases, men (n = 482/586) were predominant over women (n = 86/586), with a ratio of 5.6:1. This finding is consistent with many similar studies. ^1^^,^^3^^,^^4^^,^^11^^,^^14^^,^^15^ In our study, the majority (70%) of SD victims were from urban areas, which is consistent with other similar studies. ^4^^,^^11^ This is due to the sedentary lifestyle and westernization with the increased smoking and alcohol consumption habits adopted by people in urban areas. In addition, the stress levels of urban and rural life are well known to differ.

According to the current study, more than three-quarters of sudden deaths were unwitnessed. This could be because the shorter the survival period, the more likely it would go unnoticed, meaning that the likelihood of death being unwitnessed or unattended is directly correlated with the duration of survival.

Our study found that chest pain, dyspnea, and syncope were the most common terminal symptoms reported in 68.6% of SD victims. These are the principal symptoms of cardiovascular and respiratory diseases, which were the two leading causes of SD. The finding of chest pain as the most common prodromal symptom coincided with a study in South Africa. ^14^ In light of this, we advise emergency medical professionals to pay more attention to people who exhibit these prodromal symptoms.

The fact that 86.6% of all sudden deaths occurred outside of a hospital setting shows that the majority of these victims may not have known about their underlying medical problems or may not have been able to pay for the necessary medical care. This shows that most cases of SD are silent without preexisting symptoms. Therefore, it is vital to develop public health measures that will help educate the community about the importance of recognizing the manifestation of various clinical conditions and the need to seek immediate clinical help. Furthermore, efforts should be made to make healthcare facilities accessible and affordable with adequate diagnostic and management capacity. Two additional findings from this study further support this view. The first is that 34% of the SD victims had visits to medical facilities in the weeks before death, followed by deaths at home, which could be an indication that the diagnosis was incorrect, that therapy had failed, or that the victim lacked sufficient financial power to complete further investigation or management. The other reason is the high frequency of SD cases from chronic illnesses diagnosed for the first time at autopsy.

The occurrence of SD in males outnumbers females in all systems except GUS. Our findings coincided with those of the studies conducted in Tukey. ^4^^,^^15^ In the current study, men were predominantly affected by cardiovascular causes of sudden natural death, and a significant association was observed between CVS disease and sex. Our findings coincided with the results of the United Kingdom. ^3^^,^^10^^,^^16^ The fact that men are more frequently exposed to CVS pathologies can be attributed to the fact that estrogen hormone in women acts as a protective factor against most cardiovascular events, explaining the male preponderance that coexists with the high prevalence of CVS causes of sudden death. ^3^^,^^10^ Furthermore, it could also be the result of men's higher rates of substance misuse compared to women, as well as higher levels of financial stress.

Cardiovascular and respiratory diseases are the most common causes of SD. These results are consistent with those of various studies conducted in different parts of the world. ^1^^,^^3^^,^^4^^,^^14^^,^^15^^,^^17^ The fact that CVS pathologies are becoming the leading cause of SD could probably be the shift to the Western type of lifestyle that our societies are acquiring.

The most common underlying cause of SD is ischemic heart disease. This finding is consistent with those of various studies conducted in different parts of the world. ^1^^,^^4^^,^^5^^,^^14^^,^^15^^,^^17^ Cardiomyopathy was the second most common COD in the cardiovascular system, accounting for 5.3% of all sudden deaths. This agrees with the result achieved in Libya. ^4^

In many series, sudden cardiac death (SCD) is the initial manifestation of the condition in 20%–40% of cases. ^16^^,^^18^ In the current study, 29.8% of victims of cardiovascular death had no antecedent symptoms, and SD was the first manifestation of sudden cardiac death in 29.8% of victims, which is consistent with many similar studies. ^16^^,^^18^

Because SD is frequently the first sign of the disease, it is impossible to detect high-risk individuals, making the prevention of SCD considerably more challenging. Primary prevention is highly challenging because early identification of subjects in the community who are at a high risk of SCD is impossible owing to the significant percentage of SCD occurring in individuals with no previously known disease. However, sudden cardiac deaths have a relatively high prevalence of CVRFs. The risk of SCD in asymptomatic subjects with CVRFs is higher than that in the general population but lower than that of symptomatic patients with a diagnosed condition. ^16^

As a result, early identification and management of modifiable CVRFs is one potential strategy to reduce the burden of sudden cardiac deaths in the community. In this study, significantly higher CVRF frequencies were observed in SCD victims, including diabetes mellitus (31.7%), hypertension (17.6%), and substance addiction (22%). Therefore, community education on preventive strategies, early detection, and control of CVRFs together with preventive strategies for substance abuse would be effective for the prevention of SD.

Respiratory causes of SD accounted for 32.6% of cases in the current study. Pneumonia was the leading cause of death in the respiratory system, representing 18.7% of sudden death cases, making it the second most common cause of SD. This is closely followed by tuberculosis, comprising 6.3% of all sudden death cases. This is consistent with the results achieved in Libya and South Africa. ^4^^,^^14^

Gastrointestinal pathologies were the third leading cause of SD, occurring in 111 (19.5%) cases. Liver and pancreatic pathologies were the two most common causes of sudden death among GIS pathology, accounting for 53.2% (n=59 cases) and 37.8% (n=42 cases), respectively. Hepatic and pancreatic pathologies together contribute to 90.1% of GIS causes of sudden death and are generally the third and fourth underlying causes of death, respectively. This finding is inconsistent with various similar studies showing that CNS pathologies are the third most common cause of SD. ^3^^,^^4^^,^^14^^,^^15^^,^^17^ A possible explanation could be the high frequency of substance abuse reported among victims of GIS. A history of substance abuse was reported in 46.8% (n= 52) of 111 victims of sudden death from GIS pathology. Chronic alcohol use was the most common substance used and was reported in 50 (45%) of 111 victims, followed by khat and cigarettes in 19.8% and 4.5% of victims, respectively. Alcohol has a causative association with both liver and pancreatic diseases. ^19^^–^^22^ Additionally, there is a growing body of evidence linking khat to the emergence of both acute and chronic liver disease. ^23^^–^^26^ Khat (Catha edulis), a plant that is chewed for its psychedelic effects, is widely used in the eastern and southern parts of Ethiopia, but less frequently in the northern region. ^23^

Central nervous system pathologies accounted for 10.6% of SD cases in this study. Cerebrovascular accidents were the most common causes (81.7% of all CNS causes), followed by infectious causes (15% of all CNS causes). This is in agreement with a study from Turkey. ^15^ The number of male versus female cases in all organ systems was higher except for GUS. Our findings coincided with various studies. ^4^^,^^14^^,^^15^ The reason is that in our study, a high frequency of maternal deaths was recorded.

## Conclusions

Sudden death is an important global public health issue. Cardiovascular, respiratory and gastrointestinal system pathologies were the most common causes. The two main underlying causes of sudden death were ischemic heart disease and pneumonia. Although the causes of SD observed in the current study were comparable to those previously reported elsewhere, the rate of occurrence of certain conditions was different, particularly the higher frequency of liver and pancreatic pathologies. The association of these diseases with chronic alcohol and khat (Catha Edulis) abuse was also documented. Most of the causes of SD in Ethiopia can be prevented and treated. The majority of sudden deaths are silent without preexisting symptoms and occur outside a hospital setting. Therefore, it is vital to develop public health measures that will help educate the community about the importance of recognizing the manifestation of various clinical conditions and the need to seek immediate clinical help. Furthermore, efforts should be made to make healthcare facilities accessible and affordable with adequate diagnostic and management capacity. Documentation of autopsy-based data could provide important epidemiological information to guide medical services, prevention efforts, and control measures.

## Conflicts of interest

The authors declare that they have no conflicts of interest.

## Data Availability

Figshare: Epidemiological profiles and causes of sudden deaths of various ages in Ethiopia: an autopsy-based study, https://doi.org/10.6084/m9.figshare.24152004. ^27^ Data are available under the terms of the Creative Commons Attribution 4.0 International license (CC-BY 4.0).
