# Supplementary material for: Epidemiological profiles and causes of sudden deaths of various ages in Ethiopia: an autopsy-based study
Source: F1000Res. 2025 Feb 5;12:1441. Originally published 2023 Nov 7. [Version 2] doi: 10.12688/f1000research.142511.2 (PMC11829148; doi:10.12688/f1000research.142511.2)
Supplement: Peer review history

# Reviewer report: Reviewer response for version 2

**DOI:** 10.5256/f1000research.177058.r364673

**Published:** 2025-02-14

**Authors:** Lorraine Du Toit-Prinsloo

**Affiliations:** 1 School of Medicine and Public Health, The University of Newcastle, Callaghan, New South Wales, Australia

I am comfortable with changing my opinion to Approved

Is the work clearly and accurately presented and does it cite the current literature?

Partly

If applicable, is the statistical analysis and its interpretation appropriate?

Yes

Are all the source data underlying the results available to ensure full reproducibility?

Yes

Is the study design appropriate and is the work technically sound?

Yes

Are the conclusions drawn adequately supported by the results?

Yes

Are sufficient details of methods and analysis provided to allow replication by others?

Partly

Reviewer Expertise:

NA

I confirm that I have read this submission and believe that I have an appropriate level of expertise to confirm that it is of an acceptable scientific standard.

# Reviewer report: Reviewer response for version 1

**DOI:** 10.5256/f1000research.156070.r287008

**Published:** 2024-06-14

**Authors:** Joseph Westaby

**Affiliations:** 1 Cardiovascular and genomics research institute, St George's University of London, London, UK

The article by Lema et al, is an observational study characterising all sudden deaths observed at a single forensic centre in Ethiopia. They report sudden death was 11.5% of all cases autopsied with 5.6 times as many males than females and a mean age of 45. Cardiovascular causes were most common with ischaemic heart disease as the leading cause. They highlight the importance of epidemiological data derived from autopsy studies to inform provision of medical services.

 This is a study that adds to the literature on sudden death showing its causes from Ethiopia. There are a number of improvements that I recommend that the authors make and I would be happy to re-review the paper following their emendations.

Methods

 Individuals who died but were not confined to their homes, health facilities, or other related institutions due to illness for more than 24 hours before death were not considered unexpected deaths and were not included.

*Do the authors mean not?*

Results

 Figure 1:

*It would be best not to display the last three bars labelled as total as it does not allow you to appreciate the differences between the decades of age*

 There was a statistically significant difference between the residence of the victims and sex (p<0.001).

*It is not made clear which sex lived more commonly where. Did males more frequently live in the city or the country?*

 There was a statistically significant relationship between substance abuse and sex (p<0.001) and residence (p=0.004). A different distribution was observed for chronic alcohol use and sex (p<0.001).

*It is not made clear what these relationships are? Was it more common in males and in the city? Was alcohol more common in males or females?*

 There was a statistically significant relationship between the presence of a prior chronic illness and sex (p=0.022).

*Were males or females more likely to have prior chronic illness?*

 Table 3:

*It would be useful to put percentages for each sex for each cause so the differences between males and females are readily appreciable.*

 There was a statistically significant difference between the CVS causes and sex (p=0.019).

*Was it more common males?*

Discussion

 The findings revealed a mean age of 44.8117.349 years.

*Giving the mean age as a whole number would be more appropriate*

 Of 224 sudden death cases, men (n = 482/586) were predominant over women (n = 86/586), with a ratio of 5.6:1. This finding is consistent with many similar studies. ^1, 3, 4, 11, 14, 15^

*Only two of these studies have a ratio of 5:1, most of these have ratios of 2:1 male:female which is more usual in the literature. Could the authors please speculate as to why they have such a high male to female ratio. Are females autopsied less than males? What percentage of the 4,942 medicolegal autopsies were female?*

 The occurrence of SD in males outnumbers females in all systems except GUS. Our findings coincided with those of the studies conducted in Tukey. ^4, 15^

*Turkey is spelt wrong. They may wish to use its new name “Türkiye”. Reference four is from Libya not Turkey.*

 Our findings coincided with the results of the United Kingdom. ^3, 10, 16^

*Refences are for two studies from Nigeria and one from Spain. None are from the UK. They should include references from the UK.*

 Cardiomyopathy was the second most common COD in the cardiovascular system, accounting for 5.3% of all sudden deaths. This agrees with the result achieved in Libya. ^4^

*It would be important to say that the cardiomyopathies may be inherited and therefore it is important to screen surviving blood relatives for these conditions. (Reference-1)*

Is the work clearly and accurately presented and does it cite the current literature?

Partly

If applicable, is the statistical analysis and its interpretation appropriate?

No

Are all the source data underlying the results available to ensure full reproducibility?

Yes

Is the study design appropriate and is the work technically sound?

Yes

Are the conclusions drawn adequately supported by the results?

Yes

Are sufficient details of methods and analysis provided to allow replication by others?

Yes

Reviewer Expertise:

Cardiovascular pathology, sudden death, various cardiac conditions, anatomy of the heart

I confirm that I have read this submission and believe that I have an appropriate level of expertise to confirm that it is of an acceptable scientific standard, however I have significant reservations, as outlined above.

## References

[1] : Sudden arrhythmic death and cardiomyopathy are important causes of sudden cardiac death in the UK: results from a national coronial autopsy database. *Histopathology* .2023;82(7) : 10.1111/his.14889 1056-1066 10.1111/his.14889 36799099

# Response

**Published:** 2025-01-14

**Authors:** Alemayehu S. Lema

**Affiliations:** St. Paul's Hospital Millennium Medical College, Ethiopia

Dear Reviewer,

 We sincerely appreciate the time and effort you dedicated to providing such a comprehensive and constructive review. Your feedback has been extremely valuable in shaping the quality of this work. We have thoroughly reviewed and incorporated your suggestions throughout the revised manuscript. Please find our detailed responses to each comment below.

**Method section**

- *Response: *The statement, "Individuals who died but were not confined to their homes, health facilities, or other related institutions due to illness for more than 24 hours before death were not considered unexpected deaths and were not included," inadvertently used the phrase "not confined." This was an error; the intended criteria were that individuals that were confined. We appreciate the reviewer for identifying this inaccuracy. The statement has now been amended to read: "Individuals who died but were confined to their homes, health facilities, or other related institutions due to illness for more than 24 hours before death were not considered unexpected deaths and were not included."

**Result section**

 2.1. Figure 1 and table 3

- Following your recommendations, we have made specific modifications to the presentation of results. In Figure 1, we've removed the "total" bars to improve the visual clarity of age-related trends, enabling a better appreciation of differences across decades. In Table 3, we have included sex-specific percentages for each cause, facilitating a direct comparison of male and female distributions.
  1. Statistical data presentation
- *Response: *Thank you so much, and comments are accommodated. All the statistical analysis and interpretation of the data have been meticulously clarified and elaborated upon, offering a more robust understanding of the findings.
  1. Mean age
- The mean age (±SD) is corrected to 44.8 ± 17.349 years. The previous version incorrectly presented the standard deviation within the mean, writing it as 44.8117.349 years. This has now been rectified.

**Discussion section**

 3.1. Male: female ratio

- Response: Thank you for this critical and insightful comment. We acknowledge the discrepancy in male-to-female ratios compared to some studies in the literature. The references for this section have been appropriately amended to reflect this. We have now included a detailed discussion of potential reasons for the observed gender disparity in our study.

**Issues regarding** **inherited cardiomyopathies**

- Response: Thank you for this critical and pertinent suggestion. We agree that it is essential to highlight the potential for inherited cardiomyopathies. We have now included a statement in the discussion emphasizing the importance of screening surviving blood relatives for these conditions, and we have added the suggested reference.

**Issues regarding** **inherited cardiomyopathies**

- Response: Thank you for this critical and pertinent suggestion. We have addressed all concerns regarding the citations and references. This includes updating the reference list to ensure accurate representation of study locations and replacing older references with more current literature, where possible. Additionally, we have incorporated many recent and all suggested references.

# Reviewer report: Reviewer response for version 1

**DOI:** 10.5256/f1000research.156070.r270830

**Published:** 2024-06-10

**Authors:** Martina Modena

**Affiliations:** 1 Interdisciplinary Center for Health Sciences, Scuola Superiore Sant'Anna, Pisa, Italy

This article addresses a major health problem such as sudden death, which has a dramatic impact on families and society. The authors provide an interesting descriptive study on sudden death in Ethiopia.

 The authors should address the following comments:

 Methods:

 -    “Full postmortem examinations were carried out in each instance using the Letulle evisceration procedure […] Organs were dissected, examined and checked for signs of gross pathological changes and violence. Autopsied specimens (whole organs or organ pieces) were fixed in 10% formalin for histopathological examination. Multiple sections with thicknesses of 4-5 mm were taken. The tissues were processed, subjected to paraffin sectioning at a thickness of 4 micrometers and then stained using hematoxylin and eosin staining.” 

 The authors can provide more details on full postmortem examinations carried out? e.g. body weight and height have been collected?

 Concerning the histological analysis: was only the hematoxylin-eosin staining performed? the toxicology analysis was performed?

 The authors could include a small paragraph on heart analysis: e.g. pericardium, heart cavities, valves and coronary arteries have been checked?

 -    Considering the growing importance of molecular autopsy, have any EDTA blood or frozen tissue samples been kept for possible future analysis?

 Results:

 Causes of sudden unexpected deaths

 -    “Cardiovascular system diseases (CVS) were the leading causes of SD, accounting for 36.1% of all SD cases. This was followed by respiratory and gastrointestinal system pathologies, accounting for 32.6% and 19.5%, respectively. Central nervous system (CNS) and genitourinary system (GUS) pathologies were the least prevalent causes of death, accounting for 10.6% and 1.2%, respectively.”

 All described cases of sudden death are explained after autopsy by reaching a certain cause of death. This is surprising considering that in literature the cause of death remains in doubt even after an exhaustive autopsy in several sudden deaths. So, there is usually a proportion of cases remaining "unexplained".

 I suggest to authors to review the literature and guidelines (some references are more than 10 years old) and to review the results to be sure that all cases have been explained by autopsy. Do all cases have a definite diagnosis at autopsy? Particularly among cases of cardiac death, no probable arrhythmic death was found? Did all cases of sudden cardiovascular death have structural cardiac changes?

Is the work clearly and accurately presented and does it cite the current literature?

Partly

If applicable, is the statistical analysis and its interpretation appropriate?

Yes

Are all the source data underlying the results available to ensure full reproducibility?

Yes

Is the study design appropriate and is the work technically sound?

Partly

Are the conclusions drawn adequately supported by the results?

Yes

Are sufficient details of methods and analysis provided to allow replication by others?

Partly

Reviewer Expertise:

NA

I confirm that I have read this submission and believe that I have an appropriate level of expertise to confirm that it is of an acceptable scientific standard, however I have significant reservations, as outlined above.

# Response

**Published:** 2025-01-15

**Authors:** Alemayehu S. Lema

**Affiliations:** St. Paul's Hospital Millennium Medical College, Ethiopia

Dear Reviewer,

 We sincerely appreciate the time and effort you dedicated to providing such a comprehensive and constructive review. Your feedback has been extremely valuable in shaping the quality of this work. We have thoroughly reviewed and incorporated your suggestions throughout the manuscript. Please find our detailed responses to each comment below.

**Method section: Details of postmortem examinations procedure**

 Response: Thank you for this valuable suggestion. We have revised the manuscript to include detailed descriptions of the postmortem examinations performed, the ancillary investigations conducted, and the limitations encountered within our setting. As suggested, we have also provided a more thorough description of the gross heart examination. Your feedback has prompted us to include recommendations for improving autopsy facilities and future research, which we believe will be valuable for informing decisions and policies in similar contexts, particularly for forensic pathologists working in resource-limited facilities.

**Result section: Sudden unexplained deaths**

 Response: Thank you for this valuable suggestion. We have revised the manuscript to provide more detailed descriptions of the included cases. This study focused on all sudden unexpected deaths with a known natural cause. While our findings offer crucial epidemiological data for planning medical services, prevention, control, education, and further research, we acknowledge several limitations that could influence the interpretation of our results.

 Specifically, 6.2% of total autopsy cases remained undetermined due to the limitations of available resources and facilities, and these cases were therefore excluded from the study. Histopathological examination, largely using Hematoxylin and Eosin (H&E) staining, was performed in all sudden death cases. Toxicological, biochemical, and microbiological investigations are performed on a selective basis, as determined by the forensic pathologist. However, it is important to note that molecular autopsy facilities were not accessible, and tissue specimens were not preserved. This lack of molecular autopsy significantly limited our ability to determine the cause of death in cases of advanced putrefaction or incomplete ancillary examinations and this exclusion of cases with undetermined cause of death may affect the prevalence estimate. This limitation is explicitly discussed in the manuscript. Furthermore, we recognize that sudden unexplained deaths (SUDs) are frequently associated with underlying inherited arrhythmogenic cardiac diseases, which molecular autopsy can help identify. Identifying the cause of death via molecular autopsy is vital not only for medico-legal inquiries but also for guiding cascade genetic screening of the deceased's relatives. This screening is essential for risk prediction in family members and the appropriate management of those identified to be at risk.

 Your insightful feedback has prompted us to provide relevant recommendations regarding the need for a molecular autopsy facility. We believe that these recommendations will inform decisions and policies in our context and similar settings. As a short-term measure, considering the increasing importance of molecular autopsy, we recommend preserving DNA samples from all SUD cases to facilitate future investigations. These recommendations, we believe, will be particularly valuable for forensic pathologists and stakeholders working in resource-limited facilities.

 Despite these limitations, our study represents the first nationwide, autopsy-based investigation to comprehensively analyze the epidemiological, clinical, and pathological characteristics of all sudden unexpected natural deaths over a one-year period. We rigorously gathered information from all available sources, performed postmortem autopsies, and collected samples for available ancillary investigations. We believe that this study provides valuable epidemiological insights crucial for planning medical services, prevention, control, education, and further research.

**References**

 Response: Thank you for this critical and pertinent suggestion. We have diligently updated the references, incorporating recent publications and replacing some earlier citations with their most current versions.

# Reviewer report: Reviewer response for version 1

**DOI:** 10.5256/f1000research.156070.r244085

**Published:** 2024-02-22

**Authors:** Lorraine Du Toit-Prinsloo

**Affiliations:** 1 School of Medicine and Public Health, The University of Newcastle, Callaghan, New South Wales, Australia

I am of the opinion that this article provides a good overview of the sudden deaths admitted to a Forensic Medicine Department. 

 The following comments are to be addressed by the authors:

 Replace the word victims with deceased / decedents throughout the manuscript 

 Methods:

 - " Autopsied specimens (whole organs or organ pieces) were fixed in 10% formalin for histopathological examination. Multiple sections with thicknesses of 4-5 mm were taken. The tissues were processed, subjected to paraffin sectioning at a thickness of 4 micrometers and then stained using hematoxylin and eosin staining." - replace this with ancillary investigations included whole organ retention, histological examination of tissue slides (were there any additional investigations such as microbiology or toxicology done?)

 Circumstances of death

 - The statement referring to 36 victims died instantaneously should be further explained in the context - there are only a few causes of death in the forensic setting in my opinion where we can indicate that the death occurred instantaneously.

 Some of the references pertaining to similar studies reporting on sudden death are more than 10 years old and it would be important to obtain more recent articles if available. 

 It would also be interesting to expand on the nature of these investigations - a full post-mortem examination was conducted, but expanding on the types of ancillary investigations will add value.

Is the work clearly and accurately presented and does it cite the current literature?

Partly

If applicable, is the statistical analysis and its interpretation appropriate?

Yes

Are all the source data underlying the results available to ensure full reproducibility?

Yes

Is the study design appropriate and is the work technically sound?

Yes

Are the conclusions drawn adequately supported by the results?

Yes

Are sufficient details of methods and analysis provided to allow replication by others?

Partly

Reviewer Expertise:

Forensic Pathology - Sudden Death, Paediatric Pathology

I confirm that I have read this submission and believe that I have an appropriate level of expertise to confirm that it is of an acceptable scientific standard, however I have significant reservations, as outlined above.

# Response

**Published:** 2025-01-15

**Authors:** Alemayehu S. Lema

**Affiliations:** St. Paul's Hospital Millennium Medical College, Ethiopia

Dear Reviewer,

 We sincerely appreciate the time and effort you dedicated to providing such a comprehensive and constructive review. Your feedback has been extremely valuable in shaping the quality of this work. We have thoroughly reviewed and incorporated your suggestions throughout the manuscript. Please find our detailed responses to each comment below.

**Method section: Regarding ancillary investigations**

- Response: Thank you for this valuable suggestion. We have revised the manuscript to include detailed descriptions of the ancillary investigations performed, as well as a discussion of the available limitations within our setting. Moreover, your insightful feedback has prompted us to provide detailed recommendations for improving autopsy facilities and future research efforts, which we believe will inform decisions and policies in our and similar contexts. Histopathological examination, largely using Hematoxylin and Eosin (H&E) staining, was performed in all sudden death cases. Toxicological, biochemical, and microbiological investigations are performed on a selective basis, as determined by the forensic pathologist. However, molecular autopsy facilities were not available in our context. Despite these limitations, our study represents the first nationwide, autopsy-based investigation to comprehensively analyze the epidemiological, clinical, and pathological characteristics of all sudden unexpected natural deaths over a one-year period. We rigorously gathered information from all available sources, performed postmortem autopsies, and collected samples for available ancillary investigations. We believe that this study provides valuable epidemiological insights crucial for planning medical services, prevention, control, education, and further research.

**Circumstances of death**

- Response: Thank you for this critical and pertinent comment. We have clarified the circumstances surrounding the 6.3% (n=36/568) of all SD cases reported as "instantaneous deaths." We have further detailed that, of these 36 cases, 88.9% (n=32) were attributed to cardiovascular (CVS) causes, particularly ischemic heart disease and cardiomyopathies, while the remaining cases involved spontaneous intracranial hemorrhage.

**Terminology Changes**

- Response: Thank you for this important recommendation. We have replaced the term "victims" with "deceased" or "decedents" or Sudden death cases’’ or ‘’cases’’ throughout the manuscript as suggested.

**References **

- Response: Thank you for this critical and pertinent suggestion. We have diligently updated the references, incorporating recent publications and replacing some earlier citations with their most current versions.
